# Low-dose brain irradiation normalizes TSPO and CLUSTERIN levels and promotes the non-amyloidogenic pathway in pre-symptomatic TgF344-AD rats

**DOI:** 10.1186/s12974-022-02673-x

**Published:** 2022-12-22

**Authors:** Kelly Ceyzériat, Thomas Zilli, Philippe Millet, Nikolaos Koutsouvelis, Giovanna Dipasquale, Christine Fossey, Thomas Cailly, Frédéric Fabis, Giovanni B. Frisoni, Valentina Garibotto, Benjamin B. Tournier

**Affiliations:** 1 Division of Adult Psychiatry, Department of Psychiatry, Geneva University Hospitals, and Faculty of Medicine, Geneva University, Avenue de La Roseraie 64, 1205 Geneva, Switzerland; 2 Division of Nuclear Medicine and Molecular Imaging, Diagnostic Department, Geneva University Hospitals, and NimtLab, Faculty of Medicine, Geneva University, 1205 Geneva, Switzerland; 3 CIBM Center for BioMedical Imaging, Faculty of Medicine, University of Geneva, 1211 Geneva, Switzerland; 4 Department of Radiation Oncology, Oncology Institute of Southern Switzerland, EOC, 6500 Bellinzona, Switzerland; 5 Faculty of Medicine, University of Geneva, 1205 Geneva, Switzerland; 6 Division of Radiation Oncology, Department of Oncology, Geneva University Hospitals, 1205 Geneva, Switzerland; 7 Centre d’Études et de Recherche Sur le Médicament de Normandie (CERMN), Normandie Univ, UNICAEN, 1400 Caen, France; 8 Department of Nuclear Medicine, CHU Cote de Nacre, 1400 Caen, France; 9 Normandie Univ, UNICAEN, IMOGERE, 1400 Caen, France; 10 Institut Blood and Brain @Caen-Normandie (BB@C), Boulevard Henri Becquerel, 14074 Caen, France

**Keywords:** Radiotherapy, Alzheimer’s disease, Neuroinflammation, Amyloid, Low-dose

## Abstract

Preclinical studies have recently evaluated the impact of low-dose brain radiation therapy (LD-RT) in animal models of Alzheimer’s disease (AD) showing anti-amyloid and anti-inflammatory effects of this treatment. Its effectiveness varied, however, depending on the LD-RT protocol used and the stage when the treatment was applied. In this study, we aimed to evaluate the therapeutic potential of 10 Gy delivered in five daily fractions of 2 Gy (a protocol previously shown to induce an improvement of cognitive performances) in 9-month-old TgF344-AD rats, modeling at a pre-symptomatic stage of the disease. We showed that at an early stage, LD-RT was able to lower levels of the 18-kDa translocator protein (TSPO)-mediated neuroinflammation to normal ranges in addition to the secreted CLUSTERIN, another inflammatory protein also involved in Aβ aggregation. In addition, we demonstrated that LD-RT reduces all amyloid forms (~ − 60 to − 80%, *P* < *0.01*; soluble and aggregated forms of Aβ_40_, Aβ_42_, and Aβ_oligomers_). Interestingly, we showed for the first time that sAPPα levels were improved by the treatment, showing a higher activation of the non-amyloidogenic pathway, that could favor neuronal survival. The current evidence confirms the capacity of LD-RT to successfully modulate two pathological hallmarks of AD, namely amyloid and neuroinflammation, when applied before symptoms onset.

**Supplementary Information:**

The online version contains supplementary material available at 10.1186/s12974-022-02673-x.

## Background

Alzheimer’s disease (AD) is a devastating neurodegenerative disease mainly known for its impact on memory abilities of patients. At the molecular level, amyloid plaques and neurofibrillary tangles (NFT) are observed. The amyloid precursor protein (APP) can be cleaved by the β-secretase, releasing soluble APPβ (sAPPβ) and C99 fragments, which is cleaved by the γ-secretase, liberating pathological Aβ peptides composed by 40–42 amino acids (amyloidogenic pathway). The APP protein can also be sequentially cleaved by the α-secretase and the γ-secretase releasing the sAPPα and C83 fragments, followed by the P3 and APP intracellular domain (AICD) fragments, preventing the release of Aβ peptides (non-amyloidogenic pathway). During pathology progression, Aβ peptides aggregate and form oligomers, fibrils and ultimately amyloid plaques. Soluble forms of Aβ, including Aβ_oligomers_, are more toxic than plaques themselves [1].

Neuroinflammation is now well described as a key player in AD, and appears as an interesting target to treat Alzheimer’s disease [2]. It is characterized by morphological, transcriptomic, and functional changes of astrocytes and microglial cells that become reactive [3–5]. Activated microglia and reactive astrocytes are mostly observed around amyloid plaques [6]. However, some studies described an early increase of neuroinflammatory markers before the appearance of clinical symptoms [7, 8] and even before amyloid plaque formation [9]. The role of neuroinflammation in Alzheimer’s disease is still highly debated, and appears complex and dependent of the pathological context (amyloid alone or amyloid and tauopathy) [10, 11]. The overproduction of cytokines and chemokines such as tumor necrosis factor alpha (TNFα) or interleukins (IL), is a typical response of reactive astrocytes and activated microglial cells to a change in brain homeostasis [12]. This normal protective response become overstimulated in the case of AD, leading to a chronic and certainly deleterious inflammation with disease progression [13].

Moreover, one of the main neuroinflammation biomarker used in clinical research is the 18-kDa translocator protein (TSPO), as it is overexpressed in many brain diseases, including Alzheimer’s disease [4, 14–16]. Its cellular origin was firstly defined as purely microglial, but it is now shown that astrocytes and endothelial cells may also participate in TSPO overexpression in inflammatory conditions [17]. In AD, the astrocytic contribution to TSPO overexpression appears even before that of microglial cells [18].

Numerous studies targeting either amyloid, tau or the neuroinflammation failed to effectively stop or revert the disease process in Alzheimer’s disease patients [19]. New therapeutic strategies are constantly under development, including low-dose brain radiation therapy (LD-RT). Considered one of the standard treatments of cancer, when delivered at low- to intermediate-doses, radiotherapy has been commonly used to treat a wide range of benign conditions, including amyloid deposits and chronic inflammation diseases [20]. In the brain, different fractionation protocols of LD-RT were tested in mouse and rat models of Alzheimer’s disease at variable disease stages and post-treatment phases and the anti-amyloid and the anti-inflammatory effects of LD-RT were confirmed in most studies [21–27]. Indeed, we previously evaluated the therapeutic potential of LD-RT by comparing two different regimens (2 Gy × 5 fractions delivered weekly or daily) delivered at an advanced stage [24]. We obtained promising therapeutic effects as the memory performances in the TgF344-AD rat model significantly improved when LD-RT was delivered in five consecutive days. This effect was not accompanied by a reduction of amyloid plaques or neuroinflammation. Consequently, we hypothesized that the disease stage at which LD-RT is delivered might play an important role in the treatment response. To verify this hypothesis, we evaluated in this study the impact of the daily treatment on AD pathology when applied at pre-symptomatic stage in the TgF344-AD rat model. We quantified neuroinflammation markers, poorly and highly aggregated forms of Aβ, and for the first time we evaluated the effect of LD-RT on the non-amyloidogenic pathway.

## Methods and materials

### Animals

TgF344-AD (TgAD) rats harbor the human APP_swedish_ and PS1dE9 transgenes on a Fisher 344 background [28]. Male TgAD rats were treated at 9-month-old using radiation therapy (RT) and analyzed 1-month post-treatment (TgAD-RT, *n* = 12). Sham-treated TgAD (TgAD, *n* = 10) and sham-treated non-transgenic littermates (Wild type; WT, *n* = 10) were used as controls. Animals were housed in a 12-h light–dark cycle, with food and water ad libitum. All experimental procedures were approved by the Ethics Committee for Animal Experimentation of the Canton of Geneva, Switzerland (GE9917). All experiments complied with the Animal Research: Reporting In vivo Experiments (ARRIVE) guidelines.

### Radiation therapy

The right hemisphere of TgAD-RT rats was treated with 10 Gy delivered in five daily fractions of 2 Gy with a 6 MV direct radiation field under anesthesia (2% isoflurane) using a Truebeam® Linear Accelerator (Varian Medical Systems) as previously described [29]. Briefly, a simulation computed tomography (CT) of a rat was used to setup the half-brain irradiation using a 4-MV direct field. A 10 mm 3D-printed bolus was manufactured to avoid the presence of the build-up region in the irradiated hemisphere. Daily alignment before each treatment session was verified using a 100 kV cone beam computed tomography (CBCT) acquisition. Sham-treated animals were anesthetized daily to induce the same stress due to handling and anesthesia. Animals were analyzed 1 month after the last treatment session.

### Behavior

Animals underwent behavioral experiments before and 1 month post-treatment.

#### Alternative Y maze

The alternative Y maze test was used to assess the spatial working memory before and after LD-RT. Rats were placed at the extremity of one arm of the device (50 × 50 × 10 cm) and video-tracked for 5 min. The number of good alternation (success to the test) were automatically measured and analyzed with EthoVision software (Noldus). Rats performing less than four entries during the session were excluded.

#### Open field

The general locomotion of animals was assessed using the open field test. They were placed at the center of the square area (45 × 45 × 40 cm) and the total distance travelled was automatically measured during 1 h using a video tracking and EthoVision software.

#### Elevated plus maze

This test was used to evaluate anxiety-like behaviors. Animals were placed at the center of an apparatus, constituted by 2 open arms (anxiogenic areas; 0 × 50 × 10 cm) and 2 closed arms (50 × 50 × 10 cm), and video-tracked during 5 min. The number of head dipping over the open arms, an index inversely correlated to anxiety, was automatically measured using EthoVision software. Rats performing less than four entries during the session were excluded.

### [^125^I]-CLINDE synthesis

The CLINDE tributyltin precursor (100 µg) in acid acetic (100 µl) was incubated (70 °C, 20 min) with Na^125^I (185 MBq, PerkinElmer) and peracetic acid (37%, 5 µl). [^125^I]-CLINDE was purified using a reversed-phase column and concentrated using a Sep-Pak C18 cartridge in 95% acetonitrile (ACN). Then, ACN was evaporated and [^125^I]-CLINDE was dissolved in saline.

### Radioactivity measurement

[^125^I]-CLINDE has been synthesized following the protocol described elsewhere [17]. Animals were injected with [^125^I]-CLINDE (6.18 ± 0.15 MBq) radiotracer in the tail-vein under anesthesia (2% isoflurane) and kept anesthetized during 1 h. Then, they were euthanized, and the following regions were dissected on ice for each hemisphere separately: hippocampus, frontal cortex and the rest of the brain. Brain samples were weighted and iodine-125 radioactivity level was measured on an automatic γ counting system for each brain region. Brain concentrations were normalized to the weight of tissue of the region of interest. Samples were then snap frozen in liquid nitrogen and stored at − 80 °C until use for biochemical measurements. The blood was also collected and centrifuged at 2500*g* for 5 min to separate the plasma for biochemical measurements.

### Protein extraction

Hippocampi and frontal cortex samples were homogenized by sonication in Triton X100 lysis buffer [50 mM Tris–HCl pH = 7.4, 150 mM NaCl, 1% Triton X-100 with 1 × protease and phosphatase inhibitors (Pierce); 300 μl], centrifuged at 20,000*g* for 20 min at 4 °C. The supernatant contains Triton X100 (Tx)-soluble proteins. The pellet was re-suspended in a guanidine lysis buffer [50 mM Tris–HCl pH = 8, 5 M guanidine HCl with 1 × protease and phosphatase inhibitors (Pierce); 180 μl], incubated for 3 h on ice and centrifuged at 20,000*g* for 20 min at 4 °C. The supernatant contains guanidine (Gua)-soluble proteins.

### ELISA tests

For all ELISA tests, protein concentrations were normalized to the weight of tissue of the regions of interest.

#### Aβ measurements in hippocampus and plasma samples

Samples were diluted in diluent provided in the ELISA kit of interest: human Aβ42 ELISA test (Life Technologies), human Aβ40 ELISA test (Life Technologies), human Aβ oligomers ELISA test (IBL International). Tx-soluble samples were diluted as follow: 1/10 in the diluent buffer provide in Aβ_40_ or Aβ_oligomers_ kits, and at 1/150 in the diluent buffer for Aβ_42_. Gua-soluble samples were diluted as follow: 1/320 in guanidine buffer and then at 1/500 in diluent buffer for Aβ_42_; 1/6 in guanidine buffer and then at 1/500 in diluent buffer for Aβ_40_. For plasma, samples were diluted at 1/50 in the diluent buffer of Aβ_40_ or Aβ_42_ kits. Manufacturer’s protocols were followed. Absorbances were measured at 450 nm using the EZ read 400 microplate reader (Biochrom). The absence of amyloid in WT was validated for all forms.

#### sAPPα and sAPPβ measurements in the frontal cortex samples

Samples were diluted at 1/10 in the diluent provided in the ELISA kit of interest (Mybiosource). The procedure was performed according to the instructions of the kits. Absorbances were measured at 450 nm using the EZ read 400 microplate reader (Biochrom).

#### Inflammation markers in hippocampus and plasma samples

Brain samples were diluted in diluent provided in the kit (Cytokine & chemokine 22-plex Rat ProcartaPlex™ Panel, ThermoFisher Scientific). Brain and plasma samples were loaded in simplicates and the protocol of the manufacturer was followed. Inflammation protein concentrations were calculated using the MagPix instrument (Luminex, ThermoFisher Scientific).

### Western blot

Twenty µg of Tx-soluble proteins of the frontal cortex were denatured in 1 × Laemmli buffer, 2.5% β-mercapto-ethanol for 10 min at 70 °C and then migrated with protein ladder (All blue precision plus ladder, Biorad) in Criterion™ TGX™ precast midi protein gel (Biorad) at 150 V for 55 min with the manufacturer’s buffer (BioRad). Transfer on LF-PVDF membrane was performed for 7 min at 2.5A constant, and up to 25 V in the manufacturer buffer using the Trans-blot Turbo machine (BioRad). Membrane was saturated in 5% non-fat dry milk/TBST (20 mM Tris, 150 mM NaCl, 0.1% Tween20, pH = 7.4) for 45 min and then incubated 48 h at 4 °C with the primary antibody in 5% milk/TBST. Following 3 washes in TBST (10 min, room temperature), the membrane was immerged in the appropriated Alexa Fluor-conjugated secondary antibody in 5% milk/TBST for 90 min. Following 3 washes in TBST (10 min, room temperature), the fluorescence was detected using the iBright imaging system (ThermoFisher Scientific). The same membrane was reused after a new saturation step. Primary antibodies were used as follows: CLUSTERIN (CLU; 1/250, mouse; R&D system), GFAP-Cy3 (1/250, mouse; Sigma), GAPDH (1/5000, rabbit; Cell signaling), SERPINA3N (1/250, rabbit; Invitrogen), STAT3α (1/250, rabbit; Cell signaling) and appropriated secondary antibodies (Alexa-Fluor 488 or 555; Invitrogen) were diluted at 1/1000. Densitometry analysis was performed using ImageJ to quantify proteins and protein levels were normalized to GAPDH levels. Due to the limited number of wells on a gel, only right hemispheres of control groups (WT and sham-treated animals) were loaded. Uncropped blot images are shown in the Additional file 1.

### RNA extraction and RT-qPCR

Cortical samples were dissected on ice and placed into 400 µl of Trizol. Samples were placed at room temperature for 5 min and 400 µl of chloroform was added for 3 min. After a vortex and centrifugation at 12,000*g* for 15 min at 4 °C, the aqueous phase was collected and 1 volume of ethanol 70% was added. Samples were then transferred onto RNeasy columns (RNeasy micro kit, Qiagen) and the protocol of the manufacturer was followed. RNA was eluted with 15 µl of nuclease free water. cDNA were synthesized using the SuperScript® VILO™ cDNA synthesis kit (Life Technologies) as describe by the manufacturer. Samples were diluted at 1/10 in H2O with 100 μg/ml BSA and mixed with 250 nM of primers and Platinum SYBR-Green® (Platinum® SYBR® Green qPCR SuperMix-UDG; Life Technologies) for qPCR. The following sequences of primers were used: *Aif1-F:* CAGAGCAAGGATTTGCAGGGA*, Aif1-R:* CAAACTCCATGTACTTCGTCTTG*; Itgam-F:* CTTGGTGAAACCCGAGTGGT, *Itgam-R:* TCGATCGTGTTGATGCTACCG*; Trem2-F:* CCTGTGGGTCACCTCTAACC*, TREM2-R:* GGCCAGGAGGAGAAGAATGG*; Gfap-F:* TTGACCTGCGACCTTGAGTC*, Gfap-R:* GAGTGCCTCCTGGTAACTCG*; Vimentin-F:* AGCTGCACGATGAAGAGATCC, *Vimentin-R:* CATCCACTTCGCAGGTGAGT*; Il1β-F:* CACACTAGCAGGTCGTCATCATC, *Il1β-R:* ATGAGAGCATCCAGCTTCAAATC*; Il6-F:* AGAGACTTCCAGCCAGTTGC*, Il6-R:* AGTCTCCTCTCCGGACTTGT*; Ccl5-F:* GCAGTCGTCTTTGTCACTCG*, Ccl5-R:* GGAGTAGGGGGTTGCTCAGT; *Tnfα-F:* CAGAGCAATGACTCCAAAGTA, *Tnfα-R:* CAAGAGCCCTTGCCCTAA*; Tgf-β-F:* CCTGGAAAGGGCTCAACAC, *Tgf-β-R:* CAGTTCTTCTCTGTGGAGCTGA; *Il10-F:* GCAGTGGAGCAGGTGAAGAA, *Il10-R:* GTAGATGCCGGGTGGTTCAA*; Il4-F:* AGACGTCCTTACGGCAACAA, *Il4-R:* CACCGAGAACCCCAGACTTG; *Ppia-F:* ATGGCAAATGCTGGACCAAA, *Ppia-R:* GCCTTCTTTCACCTTCCCAAA. Expression levels of genes of interest were normalized to the abundance of *Ppia* gene with the 2^−ΔΔCt^ method.

### Statistical analysis

A sample size analysis with the graphical Douglas Altman's nomogram approach was performed [30] and significant data were reported if *P* ≤ 0.05 and *β* < 0.2. All analyses were performed in blind conditions. Normality of residues was assessed with the Shapiro–Wilk test. One-way ANOVA and Tukey’s post hoc test were used to compare plasma samples and western blot results. If data were not normal, Kruskal–Wallis test was used to compare the three independent groups, followed by Dunn’s post hoc comparisons. For other results, two-way ANOVA (Group and Hemisphere as between factors) and Tukey’s post hoc test were used to compare the groups. Outliers were identified using the ROUT method (Maximal false discovery rate = 1%). All analyses were performed on GraphPad Prism 8. Statistical details are provided in the legend of figures. Results are presented as individual values and mean ± SEM.

## Results

### Low-dose radiation therapy decreases neuroinflammation markers.

**Fig. 1:**
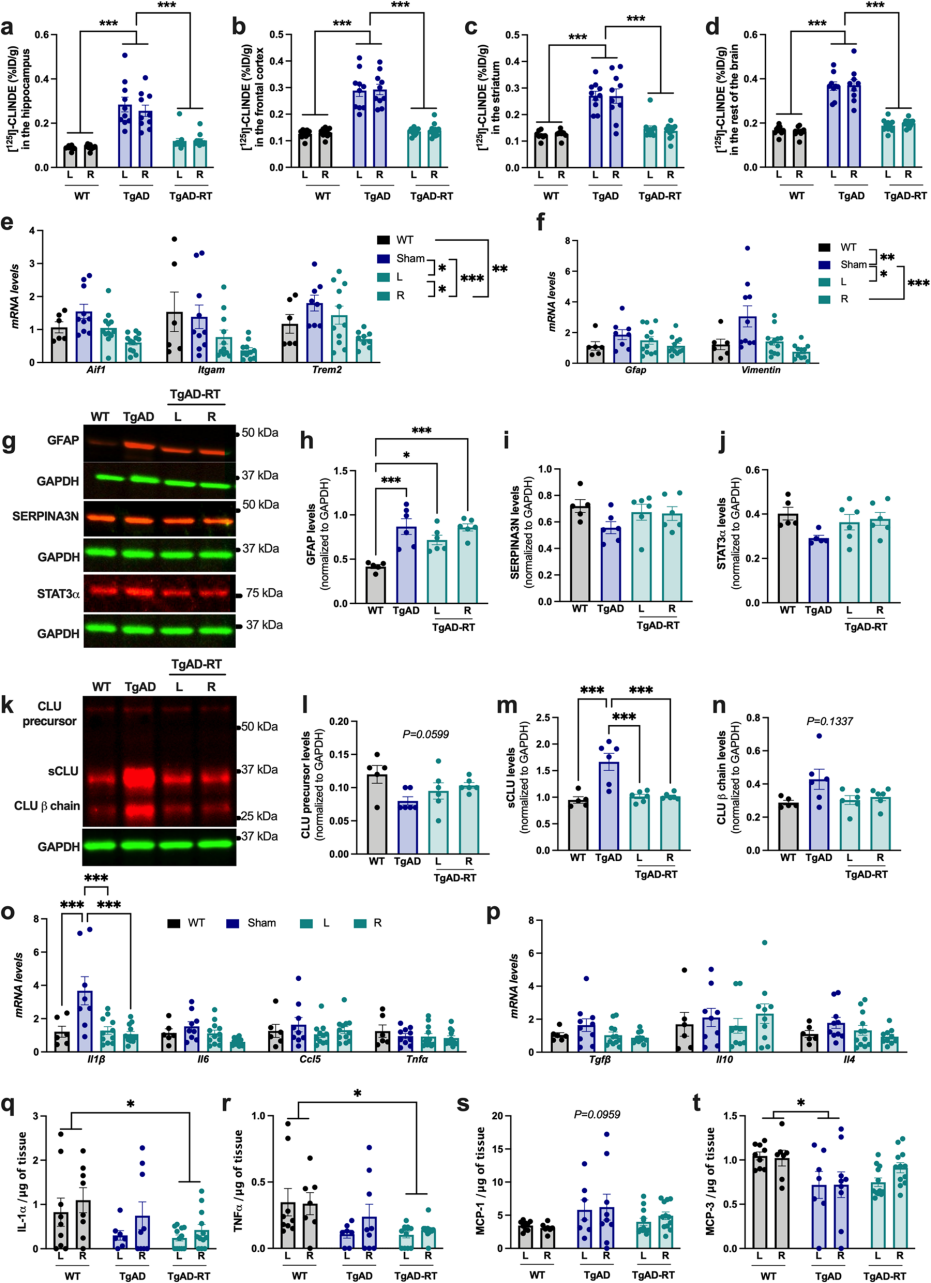
LD-RT normalizes TSPO-inflammation and decreases sCLU levels in pre-symptomatic TgAD rats. The right hemisphere of 9-month-old TgAD rats was irradiated with 10 Gy (2 Gy in 5 fractions delivered daily). *Postmortem* analyses were realized 1 month post-treatment. Ex vivo measurement of [^125^I]-CLINDE, a TSPO specific radiotracer, in the hippocampus (**a**; two-way ANOVA: *F*_(2,29)_ = 43.980, *P* < *0.0001* for group effect), the frontal cortex (**b**; two-way ANOVA: *F*_(2,29)_ = 57.90, *P* < *0.0001* for group effect), the striatum (**c**; two-way-ANOVA: *F*_(2,27)_ = 34.79, *P* < *0.0001* for group effect) or in the rest of the brain (**d**; two-way ANOVA: *F*_(2,28)_ = 77.550, *P* < *0.0001* for group effect). **e** Quantification of microglial-specific genes by qPCR in the cortex (Two-way ANOVA: *F*_(3,100)_ = 10.47, *P* < *0.0001*, for group effect). **f** Quantification of astrocyte-specific genes by qPCR in the cortex (two-way ANOVA: *F*_(3,69)_ = 7.480, *P* < *0.001* for group effect). Representing immunoblot (**g**) and quantification of GFAP (**h** One-way ANOVA, *F*_(3,19)_ = 11.72, *P* < *0.0001)*, SERPINA3N (**i**), and STAT3α (**j**) levels in the Triton-soluble fraction of the frontal cortex. **k–m** Representing immunoblot and quantification of the different forms of CLUSTERIN (CLU precursor, one-way ANOVA, *F*_(3,19)_ = 2.933, *P* = *0.0599;* secreted CLU (sCLU, one-way ANOVA, *F*_(3,19)_ = 13.91, *P* < *0.0001*) and CLUβ chain (Kruskal–Wallis test, *P* = *0.1337*) in the Triton-soluble fraction of the frontal cortex. Western blot data are normalized to GAPDH. Quantification of genes encoding for pro-inflammatory cytokines (**o**; Two-way ANOVA: *F*_(3,138)_ = 9.788, *P* < *0.0001* for group effect; *F*_(3,138)_ = 6.043, *P* < *0.001* for gene effect; *F*_(9,138)_ = 3.748, *P* < *0.001*, group x gene interaction) and anti-inflammatory cytokines (**p**; Kruskal–Wallis test) by qPCR in the cortex. **q–t** Pro-inflammatory cytokines levels quantified by ELISA in the hippocampus. Data are normalized to the weight of tissue of the region of interest. Two-way ANOVA: IL-1α: *F*_(2,24)_ = 3.392, *P* < *0.05* for group effect; TNFα: *F*_(2,27)_ = 4.918, *P* < *0.05* for group effect; MCP-1: *F*_(2,27)_ = 2.2561, *P* = *0.0959* for group effect; MCP-3: *F*_(2,27)_ = 3.988, *P* < *0.05* for group effect. Main effects or Tukey’s post hoc tests are indicated by *P < *0.05*, **P < *0.01,* ***P < *0.001.* ID: injected dose; L: left hemisphere; R: right hemisphere

Nine-month-old TgAD rats were unilaterally treated with 5 consecutive fractions of 2 Gy delivered daily. TSPO levels ([^125^I]-CLINDE binding) were significantly increased in TgAD rats compared to WT in the hippocampus (Fig. 1a), the frontal cortex (Fig. 1b), the striatum (Fig. 1c) and the rest of the brain (Fig. 1d). Interestingly, in TgAD-RT rats, we observed a restoration of TSPO levels in the irradiated hemisphere (Right hemisphere; R). This TSPO decrease was also observed in the contralateral side (Left hemisphere; L), suggesting that the effect of LD-RT on inflammation was not restricted to the targeted area.

To better understand the mechanism involved in this anti-inflammatory effect, we quantified microglial-specific genes (*Aif1, Itgam* and *Trem2;* Fig. 1e) and astrocyte-specific genes (*Gfap* and *Vimentin;* Fig. 1f) in the cortex. No increase of mRNA was observed in TgAD compared to WT rats for microglial genes, suggesting that microglial cells are not reactive yet at this age. However, LD-RT significantly reduced their expression in left and right hemispheres, with a significant higher effect in the treated hemisphere. The expression of the astrocyte-specific genes was significantly increased in TgAD compared to WT mice, showing an astrocytic reactivity. Their expression was reduced in both hemispheres, without difference between ipsi- and contra-lateral sides.

To go further, we consequently quantified astrocyte reactivity markers at the protein level by western blot in the frontal cortex of animals (Fig. 1g–n**)**. We observed an important overexpression of GFAP in TgAD rats compared to WT rats (Fig. 1g, h) but not of SERPINA3 (Fig. 1g, i) and the signal transducer and activator of transcription 3 (STAT3) (Fig. 1g, j), confirming an early but slight induction of astrocyte reactivity at this age. However, LD-RT did not impact GFAP, or the other markers, nor in the treated hemisphere or in the contralateral side.

We also quantified CLU levels by western blot, a protein expressed by astrocytes and known to be involved in Aβ aggregation (Fig. 1k–n). The CLU precursor tended to decrease in sham-treated TgAD rats compared to WT rats (Fig. 1k, l). LD-RT also tended to restore these levels. At the opposite, secreted CLU (sCLU) was increased in TgAD rats compared to WT rats (Fig. 1k, m). Interestingly, LD-RT restored normal levels of sCLU in both hemispheres. The CLU β chain tends to follow the same pattern than sCLU (Fig. 1k, n).

To measure other markers of neuroinflammation, we quantified pro- and anti-inflammatory cytokine-encoding genes. *Ilβ* expression, a gene encoding for a pro-inflammatory cytokine was increased in TgAD compared to WT rats and normalized in both hemispheres by LD-RT (Fig. 1o). All other genes were not differentially expressed between groups (Fig. 1o, p). At the protein level, 22 cytokines/chemokines were quantified in the hippocampus of rats by ELISA. All cytokines/chemokines were detectable in the hippocampus of WT and TgAD rats, excepted GM-CSF and IL-5. Interestingly, the pro-inflammatory cytokines IL-1α and TNFα were significantly decreased after LD-RT compared to WT animals (Fig. 1q, r). Monocyte chemotactic protein 1 (MCP-1) levels tend to increase in TgAD compared to WT rats, and to be restored by LD-RT (Fig. 1s). MCP-3 levels decreased in TgAD rats compared to WT rats and were restored after LD-RT (Fig. 1t). IL-2, IL-12p70, GRO-α and IL-17α, were significantly decreased in TgAD rats compared to WT rats but LD-RT did not restore normal levels *(data not shown).* Other cytokine levels were unchanged in TgAD rats compared to control animals. Cytokine/chemokine levels were also quantified in the plasma, but no difference was measured between WT and TgAD animals, nor after LD-RT.

### Low-dose radiation therapy decreases amyloid load in the hippocampus

**Fig. 2:**
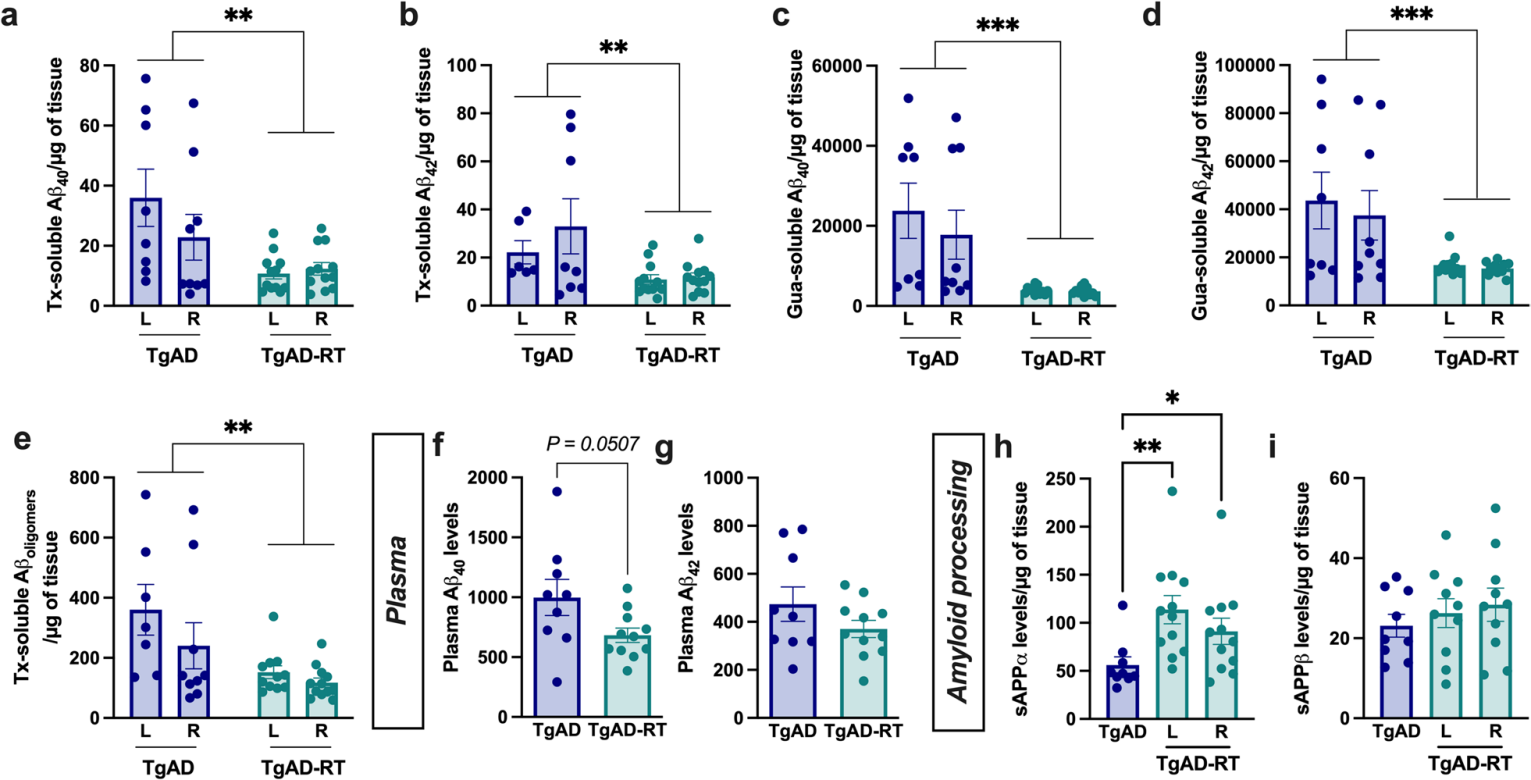
LD-RT significantly reduces amyloid load in pre-symptomatic TgAD rats. Concentration in Aβ_40_ (**a**; two-way-ANOVA: *F*_(1,37)_ = 11.40, *P* < *0.01* for the main effect of group**)** or Aβ_42_ (**b**; two-way-ANOVA: *F*_(1,34)_ = 8.681, *P* < *0.01* for the main effect of group**)** in hippocampus homogenates lysed into a Triton-X100 (Tx) buffer and analyzed by ELISA. Concentration in Aβ_40_ (**c**; Two-way-ANOVA: *F*_(1,37)_ = 19.73, *P* = *0* < *0.0001* for the main effect of group**)** or Aβ_42_ (**d**; two-way-ANOVA: *F*_(1,37)_ = 14.00, *P* < *0.001* for the main effect of group**)** in the guanidine (Gua)-soluble fraction. Concentration in Aβ_oligomers_ (**e**; two-way-ANOVA: *F*_(1,35)_ = 11.09, *P* < *0.01* for the main effect of group) measured in hippocampus homogenates lysed into a Triton-X100 (Tx) buffer. Two-way ANOVA (group, hemisphere as between factors). Concentrations in Aβ_40_ (**f**; t_(18)_ = 2.094, *P* = *0.0507*) or Aβ_42_ (**g**) in the plasma (unpaired t-test). Soluble APPα (**h**; Kruskal–Wallis, *P* < *0.01;* Dunn’s comparison) and APPβ (**i**) levels quantified by ELISA in the frontal cortex. Data are normalized to the weight of tissue of the frontal cortex. **P < 0.01, ***P < *0.001.* Hipp: hippocampus; mo: months; Gy: Gray, L: left hemisphere; R: right hemisphere

The concentration of different amyloid forms was measured in both hippocampi using ELISA tests 1 month post-treatment or sham irradiation. All Tx- and Gua-soluble forms, representing the soluble/poorly aggregated forms and the highly aggregated forms, respectively, were detectable in the hippocampus of TgAD rats at 10-month-old (Fig. 2a–e). LD-RT drastically reduced Tx-soluble Aβ_40_ (−58%, Fig. 2a) and Aβ_42_ concentration (-42%, Fig. 2b). Notably, this effect was observed in the treated hemisphere but also in the contralateral side. We also analyzed the concentrations of more aggregated forms in the Gua-soluble fractions such as fibrils and amyloid plaques. Aβ_40_ (− 81%, Fig. 2c) and Aβ_42_ concentrations (− 60%, Fig. 2d) were also reduced after treatment in both hemispheres. Moreover, the levels of Aβ_oligomers_, known to be the most toxic form in AD, were significantly reduced in LD-RT-treated rats compared to sham-treated TgAD rats (− 54%, Fig. 2e). In the plasma, LD-RT tended to also reduce Aβ_40_ (Fig. 2f) but did not impact Aβ_42_ levels (Fig. 2g).

To investigate the mechanisms involved in this Aβ reduction, we measured sAPPα and sAPPβ fragments to evaluate the effect of LD-RT on the non-amyloidogenic and the amyloidogenic pathways, respectively. LD-RT favored sAPPα levels in both hemispheres compared to sham-treated rats (Fig. 2h), without modifying the amyloidogenic pathway (Fig. 2i).

### TSPO upregulation is correlated with amyloid load in sham-treated TgAD rats

**Fig. 3:**
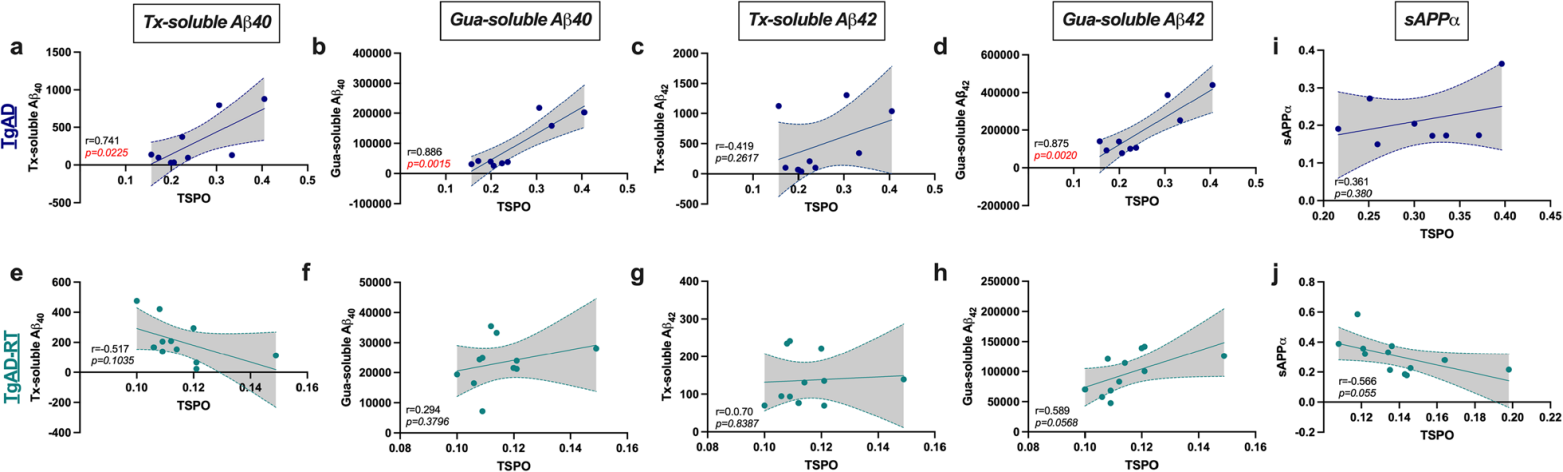
TSPO is associated with amyloid load in TgAD but not in LD-RT-treated TgAD group. Correlation (Pearson's coefficient) between TSPO levels (%ID/g) and Triton (Tx)-soluble Aβ_40_ (**a**, *r* = 0.741, *P* < *0.05;* **e**), Guanidine (Gua)-soluble Aβ_40_ **(b**, *r* = 0.886, *P* < *0.01*; **f**), Tx-soluble Aβ_42_ (**c**,** g**), Gua-soluble Aβ_42_ (**d**, *r* = 0.875, *P* < *0.01*; **h**, *r* = 0.589, *P* = *0.0568*) and sAPPα (**i**, **j**, *r* = 0.566, *P* = *0.055*) in TgAD and TgAD-RT rats

Correlation analyses revealed that the TSPO accumulation observed in TgAD was positively correlated with the amount of Tx-soluble Aβ_40_ (Fig. 3a), Gua-soluble Aβ_40_ (Fig. 3b), and Gua-soluble Aβ_42_ (Fig. 3d). However, it was not correlated with Tx-soluble Aβ_42_ concentration (Fig. 3c). Importantly, those correlations were not observed in the treated group (Fig. 3e–g), expected for Gua-soluble Aβ_42_, which almost reached significance (Fig. 3h). At the opposite, sAPPα concentration was not correlated with TSPO levels in TgAD rats (Fig. 3i) but a negative correlation, almost significant, was observed after treatment (Fig. 3j).

### Validation of the preclinical stage before treatment

An evaluation of memory performances was performed before treatment and no memory deficit was observed in TgAD rats compared to WT (Additional file 2a). Moreover, an important increase of anxiety-like behaviors was observed after LD-RT or sham treatment in all groups. Indeed, all rats explore 28.46 ± 3.32% less in the open field after than before treatment/anesthesia (Additional file 2b). A decrease of 52.31 ± 11.85% of the head dipping number was also measured (Additional file 2c). These results suggest that the increase of anxiety was not due to the irradiation but seems to be related to the repetitive anesthesia or manipulation of animals. Due to this anxiety increase, rats did not participate to the Y maze test after the treatment session, making impossible to interpret a potential effect of LD-RT on memory.

## Discussion

In this study, we evaluated the modulation of key neurochemical hallmarks of AD by a standard schedule of low-dose brain radiation therapy (LD-RT), namely 2 Gy in five fractions delivered daily, in an animal model before symptom onset. Consequently, we did not evaluate the impact of LD-RT on WT animals. A recent study showed this treatment (5 × 2 Gy daily) did not impact neuroinflammation marker (IBA1 and GFAP) levels in WT mice and did not alter cognitive performances of WT mice in the alternative Y maze test 1-, 2-, 3 or 4 weeks post-treatment [25], showing the safety of this regimen in WT animals. Moreover, we recently showed that this schedule was even able to improve memory deficits when applied at a stage with high amyloid load and neuroinflammation [24]. However, at this advanced amyloid stage, no measurable effects on amyloid plaques and inflammation were observed, possibly because of the severe pathology. Consequently, we evaluated this time the impact of this schedule at a pre-symptomatic stage. Animals were unilaterally treated at 9-month-old and AD hallmarks were assessed 1 month later.

**Fig. 4:**
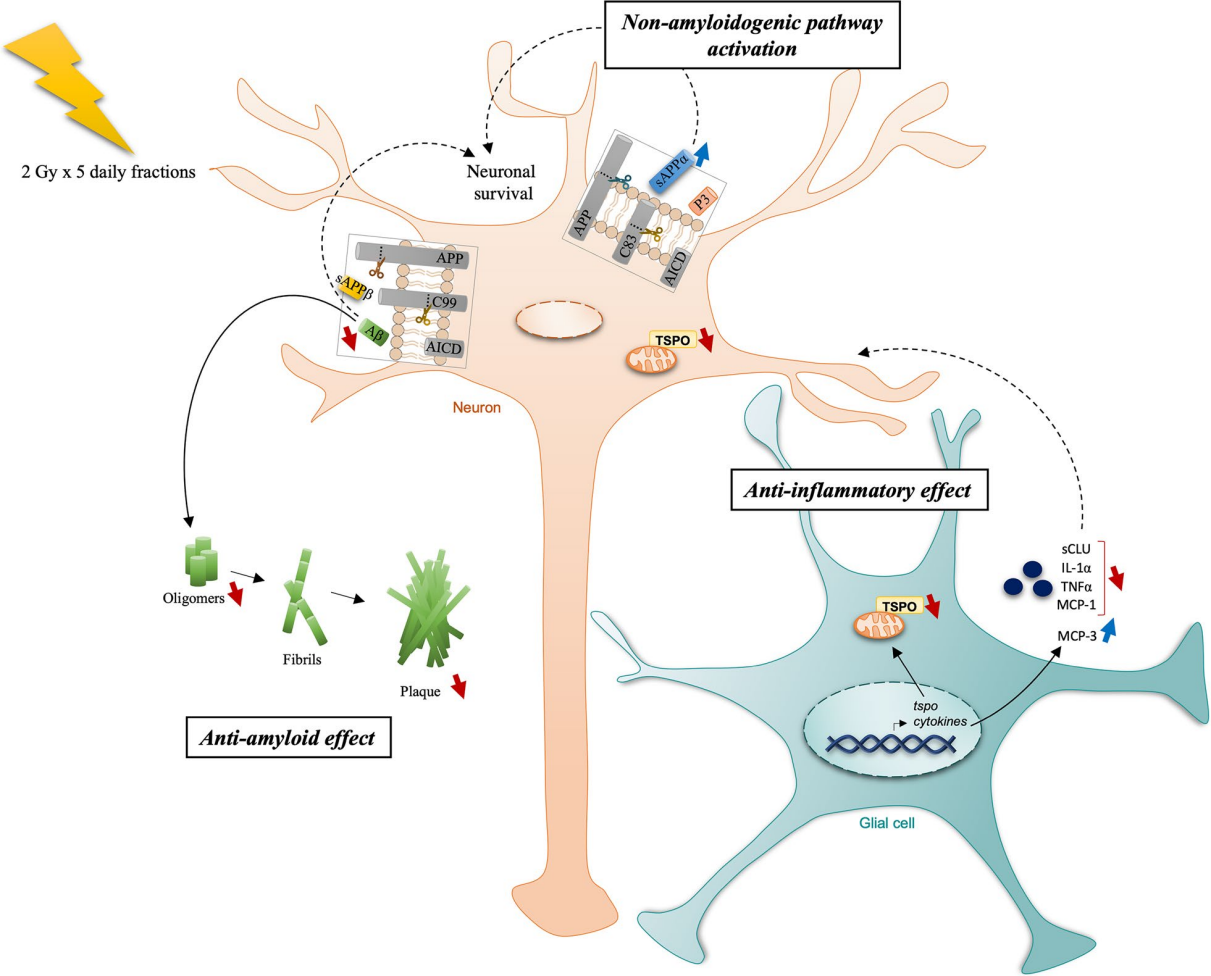
Mechanisms of LD-RT effects in the brain of pre-symptomatic TgAD rats. LD-RT treatment (10 Gy delivered in 5 daily fractions of 2 Gy) led to a drastic reduction of soluble and aggregated forms of amyloid peptides. This reduction is not due to an increase of the amyloid production. In addition, the non-amyloidogenic pathway was promoted by LD-RT. Finally, a restoration of on neuroinflammatory marker levels was measured. Altogether, those mechanisms could favor neuronal survival in TgAD rats. Solid lines indicated results demonstrated in this study. Dashed lines indicated hypothetical mechanisms

Interestingly, we observed a clear reduction of amyloid load in the hippocampus of treated TgAD rats. This decrease was measured on all forms of Aβ, namely on soluble or poorly aggregated forms (Aβ_40_, Aβ_42_ and Aβ_oligomers_), known to be the most toxic forms in AD, and on more aggregated forms such as in amyloid plaques (Fig. 4). These results are consistent with the impact of LD-RT described on peripheral amyloid pathologies (see Ceyzériat et al*.* [20] for a review) and in recent publications studying LD-RT effects in AD mouse models [21–23, 25, 26]. It is also interesting to note that the same treatment in young female 3xTg-AD mice decreased significantly highly aggregated Aβ_42_, but had only a minor effect on the other forms [26]. The different effect on soluble forms, most pronounced in male TgAD rats than female 3xTg-AD mice at the same pathological stage, could also suggest a sex-dependent response to radiation exposure. Interestingly, it has been proposed that radiosensitivity could be sex-dependent at high doses, with a higher long-term radiosensitivity in females than males [31]. However, this question has been clearly under-investigated, particularly regarding low-dose radiation exposures and further studies are necessary to investigate the impact of sex in the treatment response.

It is also important to highlight that the anti-amyloid effect we observed occurred not only in the treated hemisphere, but was also visible in the contralateral side. This result suggests a mechanism able to diffuse from the treated hemisphere to the other one considering that the out-of-field exposure to LD-RT of the contralateral hemisphere was minimal as previously investigated [29]. It could for example implicate glial cells through the modulation of the release of cytokines, that would influence Aβ production or degradation in the contralateral side [32–36]. Another mechanism involved in cell-to-cell communication could be the translocation of exosomes, known to be produced by neural cells, including astrocytes and microglia [37]. Indeed, irradiation may impact the secretion, the composition and the uptake of exosomes [38, 39], and a mechanism that may be responsible of extended bystander effects of irradiation in non-targeted area. However, the role of radiation on those modifications is still poorly understood and needs further investigation. In addition, the reduction of Aβ_40_ and the tendency of reduction of the Aβ_42_ in the plasma fraction suggests that brain load decrease was not due to a higher clearance of the amyloid in the vascular system after treatment, a mechanism known to be altered in AD [40]. For the first time, we described an increase of sAPPα levels after treatment, showing that the non-amyloidogenic pathway is promoted, without affecting the amyloidogenic pathway. The sAPPα protein having demonstrated neurotrophic and neuroprotective properties [41], its increase could directly favor neuronal survival (Fig. 4). Moreover, the negative correlation observed in the treated group suggests that the higher production of sAPPα fragment may be directly related to the decrease of TSPO-related neuroinflammation. However, the absence of modulation of sAPPβ levels shows that LD-RT does not impact Aβ production and suggests that the decrease of amyloid pathology observed after treatment is due to a modification of the degradation pathways.

The anti-inflammatory effect of irradiation at low doses is known since decades [20]. In our previous study, we did not observe this effect probably because of the too advanced amyloid pathology all over the brain. Here, we measured a restoration of basal levels of TSPO in the entire brain. This result is in accordance with our previous hypothesis and suggests that treatment should be applied in the early disease phases. Additionally, the bilateral effect also suggests that anti-inflammatory molecules diffuse from the treated hemisphere to the other one to also reduce inflammation in the contralateral side. To further evaluated this hypothesis, we quantified microglia- and astrocyte-specific genes, known to be overexpressed in inflammatory conditions. Interestingly, microglial genes were not yet changed in TgAD rats compared to WT, suggesting that microglial cells are not yet activated at this age. However, astrocytic genes were significantly increased in TgAD rats compared to WT rats, showing an early response of astrocytes compared to microglia in TgAD rats. Interestingly, LD-RT reduced microglial gene expression in both hemispheres but with a higher impact in the treated hemisphere, whereas LD-RT similarly downregulated astrocytic genes in ipsi- and contra-lateral hemispheres. In addition, we previously showed that TSPO overexpression was first linked to astrocytes in this model at 12-month-old and appeared later in microglial cells [18]. Our results and our previous observations consequently suppose that the effects of LD-RT in the contralateral side could be mediated mainly by astrocytes. We consequently evaluated the effect of LD-RT on classical markers of astrocyte reactivity, such as GFAP, SERPINA3N and STAT3 overexpression [42–45]. A clear overexpression of GFAP is indeed observed in sham-treated TgAD rats compared to WT animals, showing that astrocyte reactivity is an early phenomenon, appearing even before memory alterations and is consistent with the literature in animal models [9, 46], but also in patients [7, 8]. Surprisingly, LD-RT did not impact GFAP expression, showing that LD-RT is not able to totally deactivate astrocytes. Nevertheless, the astrocytic response in TgAD rats is slight at this age as we did not observe any upregulation of SERPINA3N and STAT3 levels or an effect of LD-RT on those markers. Interestingly, CLU (also named as Apolipoprotein J), produced and released by astrocytes, has been described has a chaperone protein for Aβ. An increase of CLU is observed in AD patients’ brains and has been shown to be early associated with the increase of the amyloid and tau pathology [47]. Dual roles of CLU have been proposed: either CLU binds to Aβ and favor its clearance through the blood brain barrier (BBB), suggesting a protective role, or it could be involved in clearance alteration and Aβ toxicity (see Foster et al*.* [48] for a comprehensive review). On the other hand, CLU deletion in mice significantly reduced amyloid pathology, showing its involvement in Aβ aggregation [49]. Moreover, it has been recently proposed that sCLU could play a central role in Aβ toxicity in human iPSC-derived neurons [50]. The restoration of basal sCLU levels observed after LD-RT is consequently consistent with the decrease of Aβ peptides but more studies should be conducted to understand if sCLU decrease is the cause or the consequence of Aβ reduction.

We also looked at cytokine/chemokine production in the brain, as astrocytes are known to overexpress cytokines when they become reactive even if the absolute level of cytokines is mainly produced by microglial cells [5], and that microglial reactivity in the hippocampus has been described only from 15-month-old in this model [51]. As expected, regarding the slight reactive state of astrocytes, only one cytokine-encoding gene was significantly increased in the TgAD rats compared to WT animals. Interestingly, the expression levels of this gene were reduced in both sides of the brain by LD-RT. At the protein level, cytokines were not increased in TgAD rats at this age. Only the pro-inflammatory MCP-1 chemokine, also known as CCL2, tended to increase in TgAD compared to WT rats and to be normalized after LD-RT. MCP-1 and its receptor CCR2 have been considerably studied in AD. An increase of MCP-1 levels in the plasma has been described in longitudinal studies in AD patients, and appears to be correlated with cognitive decline severity [52–54]. Furthermore, MCP-1 overexpression in APP transgenic mice led to an increase of amyloid plaques and memory decline, probably through a modulation microglial cells [55]. A restoration of normal MCP-1 levels after LD-RT could consequently participate in beneficial effects of LD-RT observed on Aβ and cognition at long term. Furthermore, a restoration of MCP-3 expression pattern was also observed after LD-RT and is accompanied by a reduction of two pro-inflammatory cytokines IL-1α and TNFα levels (compared to WT mice), even if not increased in TgAD rats at this age, validating the anti-inflammatory effect of LD-RT in the brain (Fig. 4).

Overall, these results suggest that TSPO decrease is an early response to LD-RT without leading to a clear deactivation of astrocytes. It opens the possibility to easily follow this effect in AD patients using TSPO positron emission tomography (PET) imaging. Remarkably, the positive correlation between TSPO levels and the global amyloid load observed in sham-treated TgAD rats is consistent with our previous finding in a mouse model of AD [56]. However, this correlation was not observed after the treatment, suggesting that anti-amyloid and anti-inflammatory mechanisms of LD-RT are two distinct effects. In other words, it supposes that TSPO reduction is not due to amyloid decrease, or vice versa and that LD-RT directly impacts inflammation and amyloid load. Nevertheless, this does not mean that the anti-inflammatory effect cannot also be involved in the degradation of amyloid through the modulation of astrocyte phagocytic properties, as an example. Further studies are necessary to decipher those mechanisms.

Finally, animals were treated at a pre-symptomatic stage as shown by the absence of alteration of behavioral performances of animals. Nevertheless, after treatment or anesthesia alone, an increase of anxiety-like behaviors was observed in all groups and treated TgAD rats did not participate to the test. This increase of anxiety has been suggested after longer exposure to isoflurane [57, 58], corroborating our hypothesis. Nevertheless, it represents a limitation of our study as it makes impossible the interpretation of LD-RT effect on memory. But due to the absence of memory alteration at this age, no improvement was expected 1 month after treatment. Longer term beneficial effects on cognition should be evaluated in future studies.

In animal models, the different stages of the pathology are well identified, making easier to choose a specific stage for applying the treatment. In addition, the TgF344-AD rat model is based on genetic mutations (as most of preclinical AD models) and are then representative of the familial AD form concerning a few percentages of AD patients (less than 5%). Our preclinical study is a proof of concept showing the clear interest of LD-RT applied in pre-symptomatic phase to decrease neuroinflammation and amyloid load, two key pathological markers observed in AD patients. Defining an equivalent pre-symptomatic at-risk condition in patients would require accurate staging using disease biomarkers and should be specifically validated. Our experiment thus paves the way for other studies which will have to evaluate the limits and effectiveness of LTD-RT, particularly according to the stages of the pathology, in addition to other doses, frequency of delivery, duration and the necessity to repeat the treatment with aging to accurately inform the design of pilot clinical studies in patients.

## Conclusions

In conclusion, our study showed that LD-RT normalized the major forms of amyloid peptides and reduced inflammation markers when the treatment was applied at a pre-symptomatic stage. Interestingly, these effects were observed on both sides of the brain, after a unilateral treatment. Our data suggest that reactive astrocytes may be the mediators of these effects, through the downregulation of cytokine production and release or through a modulation of the secreted CLUSTERIN. Overall, these data pave the way for future research to provide an in-depth characterization of the beneficial effects of LD-RT on AD. For example, the tauopathy being more related to cognitive decline, it appears important to evaluate the effect of LD-RT on this hallmark as one study showed a reduction after LD-RT, not confirmed by our group [22, 26]. Finally, the maintenance of the beneficial effects on AD markers and cognition must be validated at long term.

## Supplementary Information

**Additional file 2.** Fullblot images.

**Additional file 1.** No memory deficits in 9-months-old TgAD rats.

## Data Availability

The datasets used and/or analyzed during the current study are available from the corresponding author on reasonable request.

## Abbreviations

Aβ: Amyloid peptides
CAN: Acetonitrile
AD: Alzheimer’s disease
AICD: APP intracellular domain
APP: Amyloid precursor protein
BBB: Blood brain barrier
CLU: CLUSTERIN
IL: Interleukin
LD-RT: Low-dose brain radiation therapy
Gua-soluble: Guanidine-soluble proteins
Gy: Gray
MCP-1: Monocyte chemotactic protein 1
NFT: Neurofibrillary tangles
sAPPα/β: Soluble APP alpha or beta
sCLU: Secreted CLUSTERIN
STAT3: Signal transducer and activator of transcription 3
TgAD: TgF344-AD rat model
TNFα: Tumor necrosis factor alpha
TSPO: The 18-kDa translocator protein
Tx-soluble: Triton X100-soluble proteins

## References

[1] Mohamed A, Cortez L, de Chaves EP (2011). Aggregation state and neurotoxic properties of Alzheimer β-amyloid peptide. Curr Protein Pept Sci.

[2] Ardura-Fabregat A, Boddeke EWGM, Boza-Serrano A, Brioschi S, Castro-Gomez S, Ceyzériat K (2017). Targeting neuroinflammation to treat Alzheimer’s disease. CNS Drugs.

[3] Smith AM, Davey K, Tsartsalis S, Khozoie C, Fancy N, Tang SS (2022). Diverse human astrocyte and microglial transcriptional responses to Alzheimer’s pathology. Acta Neuropathol.

[4] Tournier BB, Tsartsalis S, Ceyzériat K, Garibotto V, Millet P (2020). In vivo TSPO signal and neuroinflammation in Alzheimer’s disease. Cells.

[5] Orre M, Kamphuis W, Osborn LM, Jansen AHP, Kooijman L, Bossers K (2014). Isolation of glia from Alzheimer’s mice reveals inflammation and dysfunction. Neurobiol Aging.

[6] Bouvier DS, Jones EV, Quesseveur G, Davoli MA, Tiago AF, Quirion R (2016). High resolution dissection of reactive glial nets in Alzheimer’s disease. Sci Rep.

[7] Carter SF, Scholl M, Almkvist O, Wall A, Engler H, Langstrom B (2012). Evidence for astrocytosis in prodromal Alzheimer disease provided by 11C-deuterium-L-deprenyl: a multitracer PET paradigm combining 11C-Pittsburgh compound B and 18F-FDG. J Nuclear Med.

[8] Rodriguez-Vieitez E, Saint-Aubert L, Carter SF, Almkvist O, Farid K, Scholl M (2016). Diverging longitudinal changes in astrocytosis and amyloid PET in autosomal dominant Alzheimer’s disease. Brain.

[9] Heneka MT, Sastre M, Dumitrescu-Ozimek L, Dewachter I, Walter J, Klockgether T (2005). Focal glial activation coincides with increased BACE1 activation and precedes amyloid plaque deposition in APP[V717I] transgenic mice. J Neuroinflamm.

[10] Ceyzériat K, Ben Haim L, Denizot A, Pommier D, Matos M, Guillemaud O (2018). Modulation of astrocyte reactivity improves functional deficits in mouse models of Alzheimer’s disease. Acta Neuropathol Commun.

[11] Guillemaud O, Ceyzériat K, Saint-Georges T, Cambon K, Petit F, Ben Haim L (2020). Complex roles for reactive astrocytes in the triple transgenic mouse model of Alzheimer disease. Neurobiol Aging.

[12] Burda JE, Sofroniew MV (2014). Reactive gliosis and the multicellular response to CNS damage and disease. Neuron.

[13] De Strooper B, Karran E (2016). The cellular phase of Alzheimer’s disease. Cell.

[14] Kreisl WC, Lyoo CH, McGwier M, Snow J, Jenko KJ, Kimura N (2013). In vivo radioligand binding to translocator protein correlates with severity of Alzheimer’s disease. Brain.

[15] Kreisl WC, Henter ID, Innis RB (2018). Imaging translocator protein as a biomarker of neuroinflammation in dementia. Adv Pharmacol.

[16] Nutma E, Ceyzériat K, Amor S, Tsartsalis S, Millet P, Owen DR (2021). Cellular sources of TSPO expression in healthy and diseased brain. Eur J Nucl Med Mol Imaging.

[17] Tournier BB, Tsartsalis S, Ceyzériat K, Medina Z, Fraser BH, Grégoire M-C (2020). Fluorescence-activated cell sorting to reveal the cell origin of radioligand binding. J Cereb Blood Flow Metab.

[18] Tournier BB, Tsartsalis S, Ceyzériat K, Fraser BH, Grégoire M-C, Kövari E (2020). Astrocytic TSPO upregulation appears before microglial TSPO in Alzheimer’s disease. J Alzheimers Dis.

[19] Ceyzériat K, Zilli T, Millet P, Frisoni GB, Garibotto V, Tournier BB (2020). Learning from the past: a review of clinical trials targeting amyloid, tau and neuroinflammation in Alzheimer’s disease. Curr Alzheimer Res.

[20] Ceyzériat K, Tournier BB, Millet P, Frisoni GB, Garibotto V, Zilli T (2020). Low-dose radiation therapy: a new treatment strategy for Alzheimer’s disease?. J Alzheimers Dis.

[21] Marples B, McGee M, Callan S, Bowen SE, Thibodeau BJ, Michael DB (2016). Cranial irradiation significantly reduces beta amyloid plaques in the brain and improves cognition in a murine model of Alzheimer’s Disease (AD). Radiother Oncol.

[22] Wilson GD, Wilson TG, Hanna A, Fontanesi G, Kulchycki J, Buelow K (2020). Low dose brain irradiation reduces amyloid-β and Tau in 3xTg-AD mice. J Alzheimers Dis.

[23] Kim S, Chung H, Ngoc Mai H, Nam Y, Shin SJ, Park YH (2020). Low-dose ionizing radiation modulates microglia phenotypes in the models of Alzheimer’s disease. Int J Mol Sci.

[24] Ceyzériat K, Zilli T, Fall AB, Millet P, Koutsouvelis N, Dipasquale G (2021). Treatment by low-dose brain radiation therapy improves memory performances without changes of the amyloid load in the TgF344-AD rat model. Neurobiol Aging.

[25] Yang E-J, Kim H, Choi Y, Kim HJ, Kim JH, Yoon J (2021). Modulation of neuroinflammation by low-dose radiation therapy in an animal model of Alzheimer’s disease. Int J Radiat Oncol Biol Phys.

[26] Ceyzériat K, Tournier BB, Millet P, Dipasquale G, Koutsouvelis N, Frisoni GB (2022). Low-dose radiation therapy reduces amyloid load in young 3xTg-AD mice. J Alzheimers Dis.

[27] Kim S, Nam Y, Kim C, Lee H, Hong S, Kim HS (2020). Neuroprotective and anti-inflammatory effects of low-moderate dose ionizing radiation in models of Alzheimer’s disease. Int J Mol Sci.

[28] Cohen RM, Rezai-Zadeh K, Weitz TM, Rentsendorj A, Gate D, Spivak I (2013). A transgenic alzheimer rat with plaques, tau pathology, behavioral impairment, oligomeric aβ, and Frank neuronal loss. J Neurosci.

[29] Koutsouvelis N, Rouzaud M, Dubouloz A, Nouet P, Jaccard M, Garibotto V (2020). 3D printing for dosimetric optimization and quality assurance in small animal irradiations using megavoltage X-rays. Z Med Phys.

[30] Ashby D. Practical statistics for medical research. Douglas G. Altman, Chapman and Hall, London, 1991. No. of pages: 611. Price: £32.00. Statistics in Medicine. 1991;10:1635–6.

[31] Narendran N, Luzhna L, Kovalchuk O (2019). Sex difference of radiation response in occupational and accidental exposure. Front Genet.

[32] Yamamoto M, Kiyota T, Walsh SM, Liu J, Kipnis J, Ikezu T (2008). Cytokine-mediated inhibition of fibrillar amyloid-β peptide degradation by human mononuclear phagocytes. J Immunol.

[33] Kiyota T, Okuyama S, Swan RJ, Jacobsen MT, Gendelman HE, Ikezu T (2010). CNS expression of anti-inflammatory cytokine interleukin-4 attenuates Alzheimer’s disease-like pathogenesis in APP+PS1 bigenic mice. FASEB J.

[34] Ghosh S, Wu MD, Shaftel SS, Kyrkanides S, LaFerla FM, Olschowka JA (2013). Sustained interleukin-1β overexpression exacerbates tau pathology despite reduced amyloid burden in an Alzheimer’s mouse model. J Neurosci.

[35] Tweedie D, Ferguson RA, Fishman K, Frankola KA, Van Praag H, Holloway HW (2012). Tumor necrosis factor-α synthesis inhibitor 3,6’-dithiothalidomide attenuates markers of inflammation, Alzheimer pathology and behavioral deficits in animal models of neuroinflammation and Alzheimer’s disease. J Neuroinflamm.

[36] Vom Berg J, Prokop S, Miller KR, Obst J, Kälin RE, Lopategui-Cabezas I (2012). Inhibition of IL-12/IL-23 signaling reduces Alzheimer’s disease-like pathology and cognitive decline. Nat Med.

[37] Budnik V, Ruiz-Cañada C, Wendler F (2016). Extracellular vesicles round off communication in the nervous system. Nat Rev Neurosci.

[38] Dai S, Wen Y, Luo P, Ma L, Liu Y, Ai J (2022). Therapeutic implications of exosomes in the treatment of radiation injury. Burns Trauma..

[39] Sun H, Sun R, Song X, Gu W, Shao Y (2022). Mechanism and clinical value of exosomes and exosomal contents in regulating solid tumor radiosensitivity. J Transl Med.

[40] Sagare AP, Bell RD, Zlokovic BV (2013). Neurovascular defects and faulty amyloid-β vascular clearance in Alzheimer’s disease. J Alzheimers Dis.

[41] Dar NJ, Glazner GW (2020). Deciphering the neuroprotective and neurogenic potential of soluble amyloid precursor protein alpha (sAPPα). Cell Mol Life Sci.

[42] Liddelow SA, Guttenplan KA, Clarke LE, Bennett FC, Bohlen CJ, Schirmer L (2017). Neurotoxic reactive astrocytes are induced by activated microglia. Nature.

[43] Ben Haim L, Ceyzeriat K, Carrillo-de Sauvage MA, Aubry F, Auregan G, Guillermier M (2015). The JAK/STAT3 Pathway Is a Common Inducer of Astrocyte Reactivity in Alzheimer’s and Huntington’s Diseases. J Neurosci.

[44] Ceyzériat K, Abjean L, Carrillo-de Sauvage M-A, Ben Haim L, Escartin C (2016). The complex STATes of astrocyte reactivity: How are they controlled by the JAK-STAT3 pathway?. Neuroscience.

[45] Bignami A, Dahl D (1976). The astroglial response to stabbing. Immunofluorescence studies with antibodies to astrocyte-specific protein (GFA) in mammalian and submammalian vertebrates. Neuropathol Appl Neuro.

[46] Chaney AM, Lopez-Picon FR, Serrière S, Wang R, Bochicchio D, Webb SD (2021). Prodromal neuroinflammatory, cholinergic and metabolite dysfunction detected by PET and MRS in the TgF344-AD transgenic rat model of AD: a collaborative multi-modal study. Theranostics.

[47] Shepherd CE, Affleck AJ, Bahar AY, Carew-Jones F, Halliday GM (2020). Intracellular and secreted forms of clusterin are elevated early in Alzheimer’s disease and associate with both Aβ and tau pathology. Neurobiol Aging.

[48] Foster EM, Dangla-Valls A, Lovestone S, Ribe EM, Buckley NJ (2019). Clusterin in Alzheimer’s disease: mechanisms, genetics, and lessons from other pathologies. Front Neurosci.

[49] DeMattos RB, O’dell MA, Parsadanian M, Taylor JW, Harmony JAK, Bales KR (2002). Clusterin promotes amyloid plaque formation and is critical for neuritic toxicity in a mouse model of Alzheimer’s disease. Proc Natl Acad Sci U S A.

[50] Robbins JP, Perfect L, Ribe EM, Maresca M, Dangla-Valls A, Foster EM (2018). Clusterin is required for β-amyloid toxicity in human iPSC-derived neurons. Front Neurosci.

[51] Voorhees JR, Remy MT, Erickson CM, Dutca LM, Brat DJ, Pieper AA (2019). Occupational-like organophosphate exposure disrupts microglia and accelerates deficits in a rat model of Alzheimer’s disease. NPJ Aging Mech Dis.

[52] Bettcher BM, Neuhaus J, Wynn MJ, Elahi FM, Casaletto KB, Saloner R (2019). Increases in a pro-inflammatory chemokine, MCP-1, are related to decreases in memory over time. Front Aging Neurosci..

[53] Lee W-J, Liao Y-C, Wang Y-F, Lin I-F, Wang S-J, Fuh J-L (2018). Plasma MCP-1 and cognitive decline in patients with Alzheimer’s disease and mild cognitive impairment: a two-year follow-up study. Sci Rep.

[54] Sanchez-Sanchez JL, Giudici KV, Guyonnet S, Delrieu J, Li Y, Bateman RJ (2022). Plasma MCP-1 and changes on cognitive function in community-dwelling older adults. Alzheimer’s Res Ther.

[55] Kiyota T, Yamamoto M, Xiong H, Lambert MP, Klein WL, Gendelman HE (2009). CCL2 accelerates microglia-mediated Abeta oligomer formation and progression of neurocognitive dysfunction. PLoS ONE.

[56] Tournier BB, Tsartsalis S, Rigaud D, Fossey C, Cailly T, Fabis F (2019). TSPO and amyloid deposits in sub-regions of the hippocampus in the 3xTgAD mouse model of Alzheimer’s disease. Neurobiol Dis.

[57] Toyama K, Spin JM, Abe Y, Suzuki Y, Deng AC, Wagenhäuser MU (2019). Controlled isoflurane anesthesia exposure is required for reliable behavioral testing in murine surgical models. J Pharmacol Sci.

[58] Hohlbaum K, Bert B, Dietze S, Palme R, Fink H, Thöne-Reineke C (2017). Severity classification of repeated isoflurane anesthesia in C57BL/6JRj mice-assessing the degree of distress. PLoS ONE.

