# Supplementary material for: Low-dose brain irradiation normalizes TSPO and CLUSTERIN levels and promotes the non-amyloidogenic pathway in pre-symptomatic TgF344-AD rats
Source: J Neuroinflammation. 2022 Dec 22;19:311. doi: 10.1186/s12974-022-02673-x (PMC9783748; doi:10.1186/s12974-022-02673-x)
Supplement: Supplementary file 1 — Additional file 2. Fullblot images. [file 12974_2022_2673_MOESM1_ESM.docx]

**
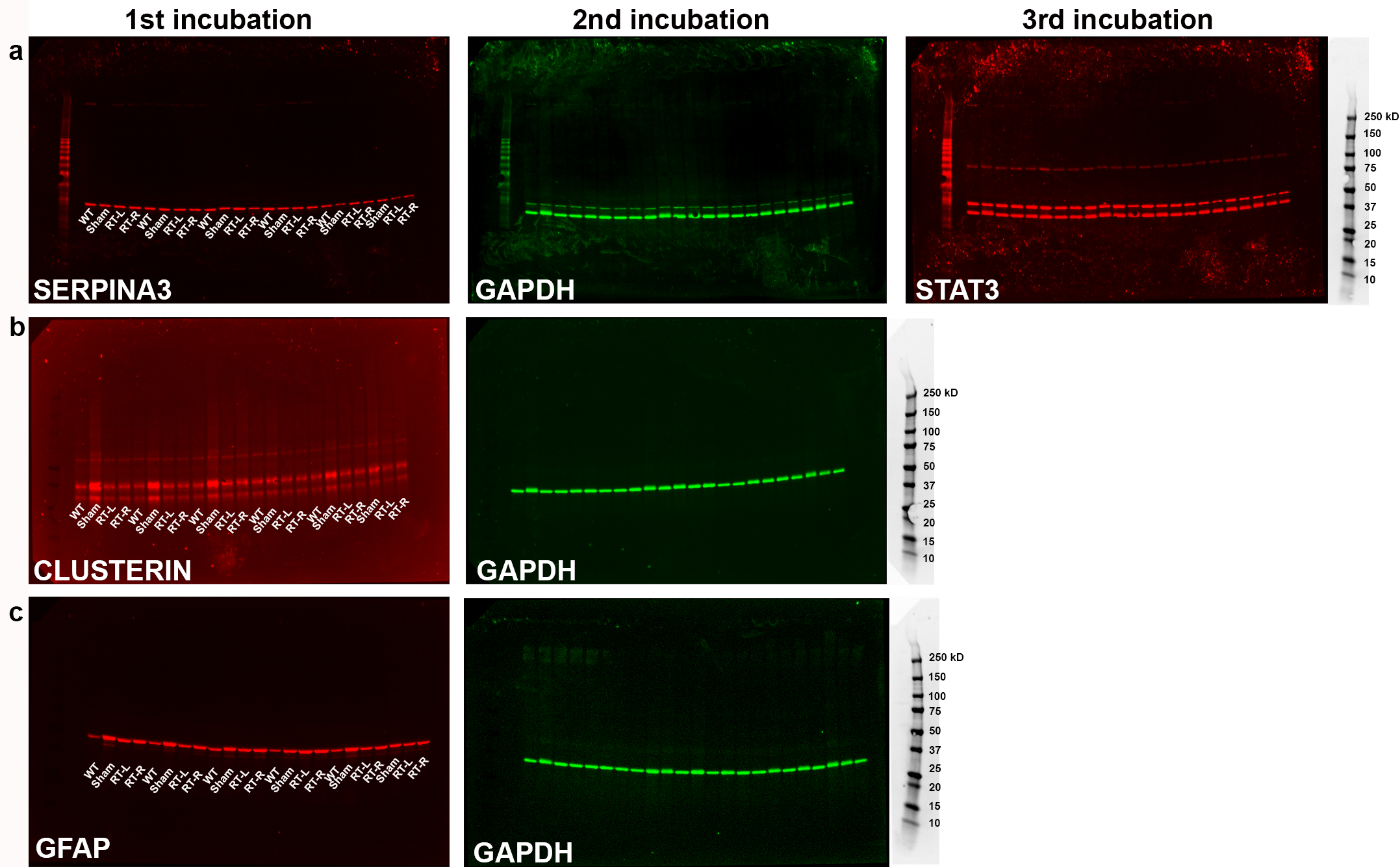
Additional File 1. Full blot images.**

**(a)** Full images used for the quantification of SERPINA3, GAPDH and STAT3α protein levels. **(b)** Full images used for the quantification of CLUSTERIN and GAPDH protein levels. **(c)** Full images used for the quantification of GFAP and GAPDH protein levels.
