# Supplementary material for: Low-dose brain irradiation normalizes TSPO and CLUSTERIN levels and promotes the non-amyloidogenic pathway in pre-symptomatic TgF344-AD rats
Source: J Neuroinflammation. 2022 Dec 22;19:311. doi: 10.1186/s12974-022-02673-x (PMC9783748; doi:10.1186/s12974-022-02673-x)
Supplement: Supplementary file 2 — Additional file 1. No memory deficits in 9-months-old TgAD rats. [file 12974_2022_2673_MOESM2_ESM.docx]

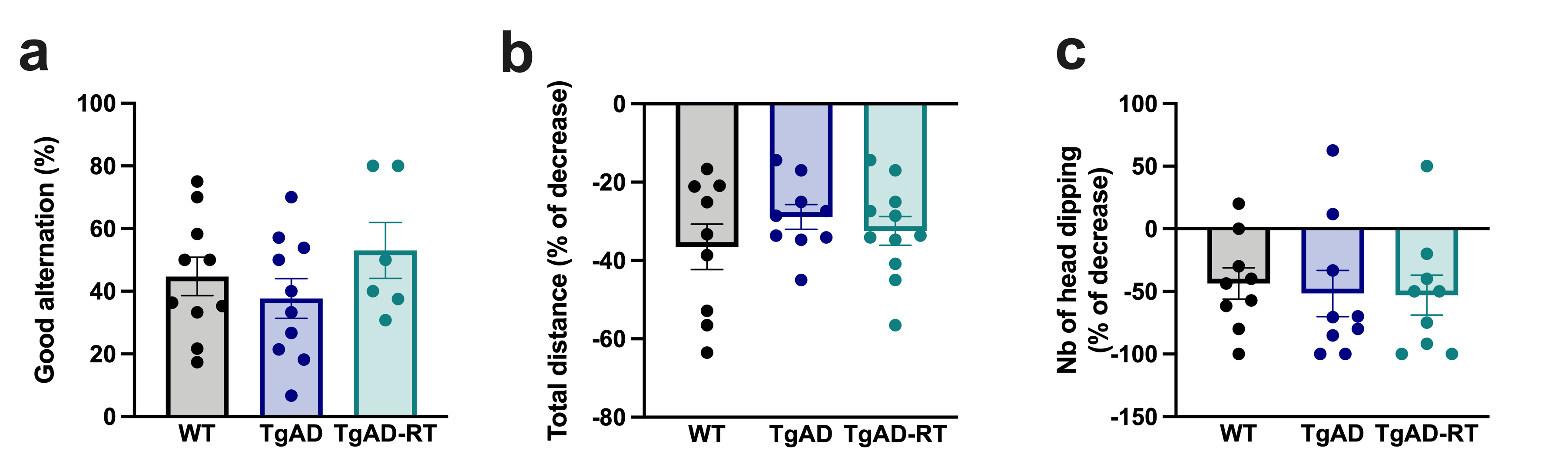


**Additional file 2: No memory deficits in 9-months-old TgAD rats.**

**a)** Working memory performances of animals before LD-RT at the Y maze test. **b)** Total distance travelled in the open field apparatus after LD-RT or anesthesia compared to pre-treatment behavior. **c)** Number of head dipping in the open arms of the elevated plus maze after LD-RT or anesthesia compared to pre-treatment behavior. One-way ANOVA.
